# Egr-1 regulates irradiation-induced autophagy through Atg4B to promote radioresistance in hepatocellular carcinoma cells

**DOI:** 10.1038/oncsis.2016.91

**Published:** 2017-01-30

**Authors:** W-x Peng, Y-y Wan, A-h Gong, L Ge, J Jin, M Xu, C-y Wu

**Affiliations:** 1 Department of Oncology, the Affiliated People's Hospital of Jiangsu University, Zhenjiang, Jiangsu, China; 2 Department of Cell biology, School of Medicine, Jiangsu University, Zhenjiang, China; 3 Department of Gastroenterology, Affiliated Hospital of Jiangsu University, Zhenjiang, Jiangsu, China

## Abstract

Although hepatocellular carcinoma (HCC) is usually response to radiation therapy, radioresistance is still the major obstacle that limits the efficacy of radiotherapy for HCC patients. Therefore, further investigation of underlying mechanisms in radioresistant HCC cells is warranted. In this study, we determined the effect of early growth response factor (Egr-1) on irradiation-induced autophagy and radioresistance in HCC cell lines SMMC-7721 and HepG2. We showed that autophagy-related gene 4B (Atg4B) is induced by Egr-1 upon ionizing radiation (IR) in HCC cells. Luciferase reporter assays and chromatin immunoprecipitation (ChIP) revealed that Egr-1 binds to the Atg4B promoter to upregulate its expression in HCC cells. Suppression of Egr-1 function by dominant-negative Egr-1 dampens IR-induced autophagy, cell migration, and increases cell sensitivity to radiotherapy. Together, these results suggest that Egr-1 contributes to HCC radioresistance through directly upregulating target gene Atg4B, which may serve as a protective mechanism by preferential activation of the autophagy.

## Introduction

Hepatocellular carcinoma (HCC) is recognized as the most prevalent and aggressive primary liver malignancy.^1^ Most patients miss the best time window of surgery or liver transplantation as they are often diagnosed at middle and late stage.^2, 3^ Thus, radiotherapy and chemotherapy are especially important to HCC treatment. Although HCC is initially responsive to radiation therapy well, the development of radioresistance is almost inevitable.^4, 5^ Therefore, understanding of the molecular mechanism of radioresistance is critical to overcome the resistance.

Autophagy, the major intracellular pathway for the degradation of protein, has been shown to play a protective role for the anticancer treatment by removing the damaged protein.^6, 7^ Moreover, accumulating evidence indicates that autophagic response of cancer cells to ionizing radiation (IR) may have a major role on cellular survival.^8, 9, 10, 11^ For instance, the induction of autophagy by IR contributes to cell survival of glioma cells.^12^ Knockdown of autophagy-related genes (Atg) 4B, Atg5 and Atg12 by RNAi results in retardation of DNA double-strand breaks repair, and thus, leads to radiosensitization.^13^ Further studies have shown that autophagy inhibitors, 3-methyladenine (3-MA) and chloroquine (CQ), significantly increase the radiosensitivity of the radioresistant MDA-MB-231 cell line.^9, 14^ Although, many recent reports indicate the protective role of autophagy in IR exposure, the detailed underlying mechanisms are still elusive.

Early growth response factor (Egr-1), an immediate early gene and a zinc finger transcription factor, is rapidly induced in response to IR.^15, 16, 17^ Upon irradiation, Egr-1 can act as a master transcription factor that controls the expression and regulation of various proteins, and other transcription factors to inhibit apoptosis and enhance tumor growth.^18, 19, 20^ Our previous studies showed that Egr-1 promotes hypoxia-induced autophagy to enhance chemoresistance of HCC cells.^21^

Although IR-induced upregulation of Egr-1 and autophagy have been implicated in cancer radioresistance, the precise role of Egr-1 and autophagy in this aspect especially in HCC remain unclear. Thus, the present study, built upon previous findings, aimed to determine the role of Egr-1 in radioresistance of HCC cells. We showed that Egr-1 transcriptionally activates Atg4B, and facilitates IR-induced autophagy. Furthermore, this Egr-1/Atg4B signaling axis regulates radioresistance of HCC cells.

## Results

### Egr-1 promotes radioresistance in HCC cells

**Figure 1:**
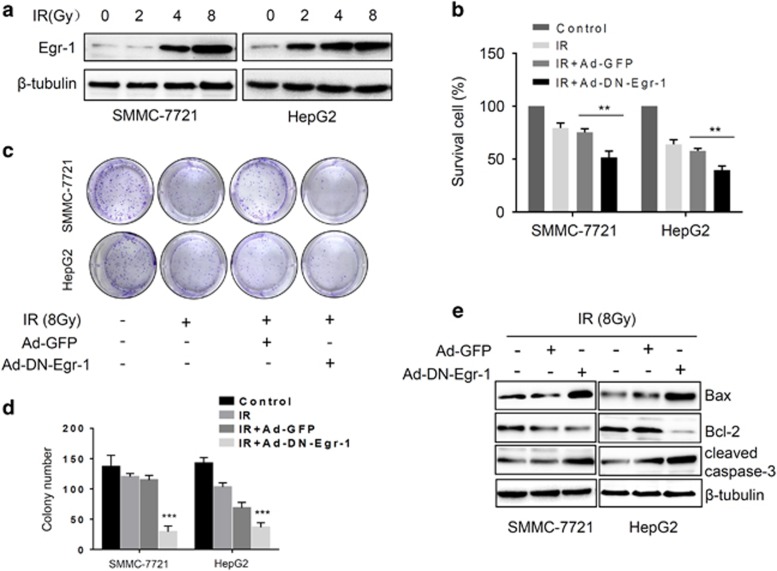
Egr-1 promotes radioresistance in HCC cells. (**a**) Egr-1 expression was rapidly induced by radiation treatment. Western blot analysis of Egr-1 expression after different doses of IR treatment. (**b**) Survival of cells was examined by CCK-8 assay. SMMC-7721 and HepG2 cells were infected with Ad-GFP or Ad-DN-Egr-1 followed by IR (8 Gy) treatment, **P<0.01 vs Ad-GFP group. (**c**, **d**) Suppression of Egr-1 transcription activity inhibits colony-formation abilities of HCC cells, ***P<0.001 vs Ad-GFP group. (**e**) Suppression of Egr-1 transcription activity enhances IR- induced apoptosis.

Recent evidence shows that Egr-1 can be rapidly induced by IR and protects cancer cells from IR-induced cell death by regulation of apoptotic-related genes Bax, p53 and AIF in glioma and colorectal cancer cell lines.^22, 23^ To obtain the insight into the role of Egr-1 in HCC cells upon IR exposure, we determined Egr-1 expression in response to different IR doses in HepG2 and SMMC-7721 cells. Western blot revealed that Egr-1 was significantly induced in cells receiving 8 Gy irradiation (Figure 1a). In consideration of previously reported anti-apoptotic function of Egr-1 upon IR, we asked whether the increased Egr-1 expression contributes to radioresistance of HCC cells. Thus, we infected SMMC-7721 and HepG2 cells with adenovirus delivered vector control (Ad-GFP) and dominant-negative Egr-1 (Ad-DN-Egr-1) as described previously.^21^ A significantly decrease of cell viability was detected after 8 Gy irradiation exposure in Ad-DN-Egr-1 infected group verse the vector control group (Figure 1b). In response to IR (8 Gy), the respective levels of survival cells at 72 h were 74.9% in control group and 49.4% in Ad-DN-Egr-1 infected group in SMMC-7721 cells and the percentages are 61.3% and 38.2% in HepG2 cells, respectively. To further analyze the radioresistance ability of Egr-1, we used colony-formation assay to assess survival of HCC cells after IR exposure. Our results showed a dramatic decrease in clonogenic growth after IR in Ad-DN-Egr-1 infected group compared with vector control group (Figure 1c and d). Meanwhile, we attempted to determine the role of Egr-1 on IR-induced apoptosis, the expression of apoptosis marker gene Bcl-2, Bax and cleaved caspase-3 were analyzed by western blot. As shown in Figure 1e, IR decreased the expression of anti-apoptotic protein Bcl-2, and increased the expression of apoptotic protein Bax and cleaved caspase-3, simultaneously. Collectively, these results suggested that Egr-1 promotes the radioresistance of HCC cells.

### Egr-1 promotes migration ability of HCC cell upon IR treatment

**Figure 2:**
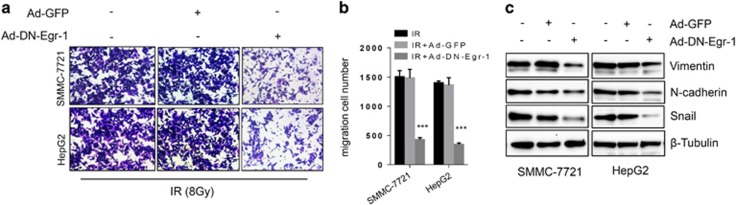
Egr-1 promotes cell migration upon IR. (**a**, **b**) Suppression of Egr-1 attenuates migration capability of HCC cells after IR treatment. (**a**) Photography and (**b**) quantitative chart of the transwell assay without matrigel coated was employed to detect the cell migration ability in SMMC-7721 and HepG2 cells infected with Ad-GFP or Ad-DN-Egr-1, ***P<0.001 vs Ad-GFP group. (**c**) Depletion of Egr-1 by Ad-DN-Egr-1 significantly inhibited the expression of Vimentin, N-Cadherin and Snail in SMMC-7721 and HepG2 cells determined by western blots analysis.

Several recent studies suggested that radiotherapy can promote tumor invasion and metastasis in various types of cancer.^24, 25, 26^ To investigate the effect of Egr-1 on migration ability of HCC cells upon IR, we performed transwell assay of HCC cells infected with Ad-GFP or Ad-DN-Egr-1. A significant increase (approximately fourfold) in the migration potential of Ad-DN-Egr-1 was seen (Figure 2a and b), suggesting critical roles of Egr-1 in enhanced migration potential of HCC cells upon IR. To determine the molecular mechanism of Egr-1 facilitated cell motility, we tested some cell migration protein markers. As shown in Figure 2c, suppression of Egr-1 activity decreased the IR-induced upregulation of vimentin, *N*-cadherin and snail protein level compared with vector control. These results suggested that Egr-1 promotes HCC cells migration upon IR treatment.

### Radiation-induced autophagy confers resistance to radiotherapy in HCC cells

**Figure 3:**
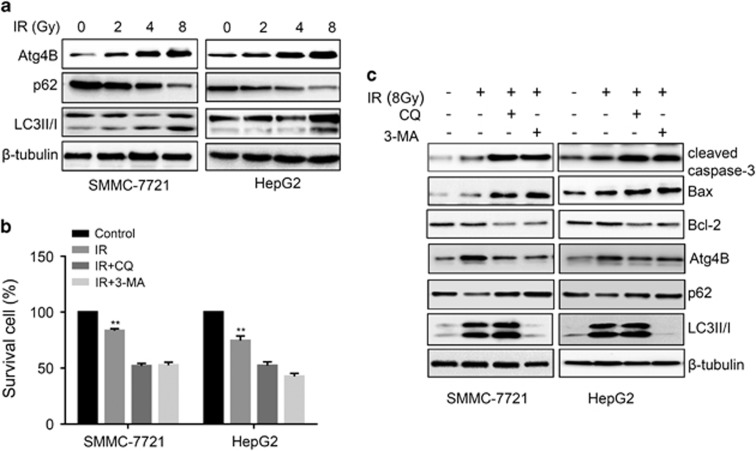
IR-induced autophagy facilitates radioresistance of HCC cells. (**a**) IR treatment induces autophagy as dose-dependent manner. The expression of autophagic marker Atg4B, LC3 and p62 were detected by western blot. (**b**) Inhibition of autophagy decreases cell viability after IR treatment. SMMC-7721 and HepG2 cells were pre-treated with 3-MA (2 mm) and CQ (30 μM) for 24 h, then cells were exposure to IR at 8 Gy. Cell viability of cell was measured by CCK-8 72 h after IR treatment. (**c**) Depletion of autophagy promotes IR-induced cell apoptosis. SMMC-7721 and HepG2 cells were pre-treated with 3-MA (2 mM) and CQ (30 μm) for 24 h, then cells were exposure to IR at 8 Gy. The expression of apoptotic markers cleaved caspase-3, Bax, Bcl-2 and autophagy markers Atg4B, p62 and LC3 were explored by western blot.

Increasing evidence indicates that the induction of autophagy contributes to resistance to anticancer treatments.^27, 28^ We hypothesized that HCC cells may exhibit increased autophagy after IR exposure. Thus, we determined expression of autophagic markers after irradiation exposure by western blot. As shown in Figure 3a, compared with untreated group, radiation increased the level of Atg4B, LC3II and decreased the level of autophagy substrate protein p62/ SQSTM1 in SMMC-7721 and HepG2 cells, especially at 8 Gy. To determine the function of radiation-induced autophagy in HCC cells, we utilized the autophagy inhibitors 3-MA and CQ to block autophagy and then examined whether radiation-induced autophagy impacts proliferation of HCC cells. We found that HepG2 and SMMC-7721 cells with autophagy depletion proliferated much more slowly than control cell did after IR exposure (Figure 3b). Consistent with this result, immunoblotting analysis showed that the level of Bcl-2 remarkably decreased, but the expression of Bax and cleaved caspase-3 was increased in CQ and 3-MA group (Figure 3c). Taken together, these results suggested that autophagy promotes resistance to radiotherapy of HCC cells.

### Atg4B is a direct transcriptional target of Egr-1

**Figure 4:**
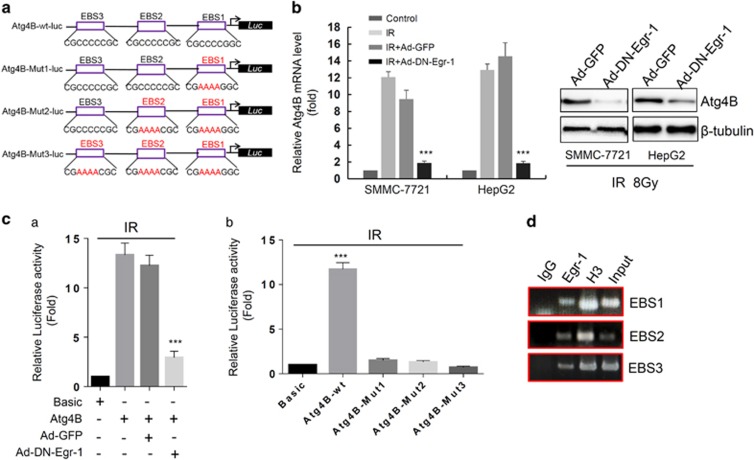
Egr-1 directly binds to Atg4B promoter. (**a**) Schematic description of three potential Egr-1 binding sites and the mutation sites on the human Atg4B promoter. (**b**) Egr-1 regulates Atg4B expression. Atg4B mRNA and protein level were measured by qPCR and western blot, respectively. (**c**) Atg4B promoter activity under stimulation by IR exposure. (**a**) SMMC-7721 were infected with Ad-GFP or Ad-DN-Egr-1. (**b**) Wild-type or mutant Atg4B promoter constructs were transfected into SMMC-7721 cells along with different combinations of expression vectors and/or their vector. (**d**) Identification of Egr-1 binding to Atg4B promoter *in vivo* by ChIP assay. Lysates from SMMC-7721 cells were subjected to ChIP by using ChIP assay kit from CST. Sonicated chromatin was used as input, Histone H3 antibody was used as positive control, and IgG was used as negative control (IgG). The band of ChIP-PCR products amplified by Atg4B-ChIP primers were shown.

The observation of increased Egr-1 expression and induced autophagy after IR led us to hypothesize that Egr-1 may decrease the sensitivity of HCC cells to IR. Cysteine protease Atg4B is required for cleaving the C-terminal amino acid of LC3, which is essential for LC3 protein conjugation to phosphatidylethanolamine and insertion to membranes. This step is necessary for autophagy.^29, 30^ Sequence analysis using Tfsitescan (IFTI-MIRAGE website,^31^ revealed that the 766 bp proximal fragment corresponding to Atg4B contains three potential EBS (Egr-1 binding site), to which we will refer from now on as site 1 (sequence: 5′-CGCCCCGGC-3′, location: −309 to −301), site 2 (sequence: 5′−-CGCCCCCGC-3′, location: −428 to −420 bp), site 3 (sequence: 5′-CGCCCCCGC-3′, location: −767 to −759 bp) (Figure 4a). This suggests the possibility that Atg4B gene expression may be regulated through the interaction of the Egr-1 protein with the Atg4B promoter. To investigate the above hypothesis, we next studied the effects of Egr-1 activity on the expression of Atg4B in SMMC-7721 and HepG2 cells. As expected, suppression of Egr-1 activity caused downregulation of Atg4B at the message and protein levels (Figure 4b). Thus, we cloned the putative human Atg4B promoter in a luciferase reporter. Luciferase assays revealed that Egr-1 was critical to Atg4B promoter activity through binding to region (−767 to −301) under stimulation by IR exposure because the mutations in these binding sites exhibited lower promoter activity (Figure 4c). To further determine the regulation of Atg4B by Egr-1, we performed chromatin immunoprecipitation (ChIP) analysis. All of three Egr-1-binding sites of the Atg4B promoter were interacted with Egr-1 protein in SMMC-7721 cells (Figure 4d). These results suggested that Atg4B is a direct transcriptional target of Egr-1 by interacting with the Atg4B promoter at Egr-1 binding sites.

### Egr-1 promotes resistance to IR by enhancing irradiation-induced autophagy

**Figure 5:**
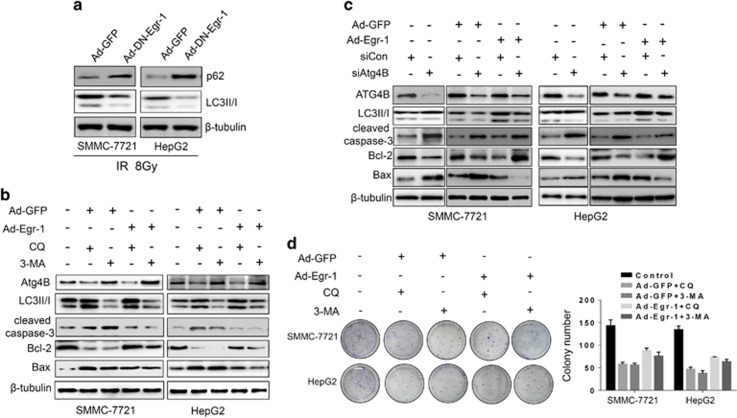
Egr-1 promotes HCC Cells resistance to IR by facilitating irradiation-induced autophagy. (**a**) Repression of Egr-1 attenuates IR-induced autophagy. Cells were infected with Ad-GFP or Ad-DN-Egr-1. Western blot examines the expression of p62 and LC3 after IR (8 Gy) treatment. (**b**, **c**) Overexpression Egr-1 restores cell apoptosis caused by inhibition of autophagy by 3-MA, CQ or Atg4B siRNA. Cells were infected by Ad-GFP or Ad-Egr-1, followed 3-MA (2 mM), CQ (30 μm) treatment (**b**) or siRNA (100 nM) transfection. (**c**) The expression of apoptotic markers C-caspase-3, Bax, Bcl-2 and autophagy markers Atg4B and LC3 were detected by western blot. (**d**) Overexpression of Egr-1 increases colony-formation ability of HCC cells. Photography and graph indicates colony numbers of each experimental group.

To determine whether Egr-1 regulates Atg4B to promote autophagy-induced radioresistance of HCC cells, we transfected SMMC-7721 and HepG2 cells with Ad-GFP or Ad-DN-Egr-1. We then treated the cells with 8 Gy radiation 48 h after infection, and examined the biomarkers of autophagy and apoptosis by western blot. As shown in Figure 5a, suppression of Egr-1 activity decreased the expression of LC3II/LC3I ratio, and thus in turn increased the p62/SQSTM1 expression as compared with vector control. To further investigate the function of autophagy on Egr-1 induced radioresistance, we infected SMMC-7721 and HepG2 cells with Ad-GFP or Ad-Egr-1, followed by autophagy inhibitor treatment and Western blot. As shown in Figure 5b, abrogation of autophagy increased the expression of cleaved caspase-3 and Bax, whereas it decreased the protein level of Bcl-2. Inhibition of autophagy by siRNA targeting Atg4B also supported that autophagy contributes to Egr-1 facilitated radioresistance in HCC cells (Figure 5c). In addition, we also investigated the effect of autophagy on Egr-1 promoted clonogenic growth of HCC cells. As shown in Figure 5d, clonogenic assay clearly showed that clone formation of HCC cells was reduced after exposure to 3-MA or CQ. However, overexpression of Egr-1 by Ad-Egr-1 restores the inhibition of clonogenic growth by autophagy inhibitor.

### Atg4B is overexpressed in human HCC specimens

**Figure 6:**
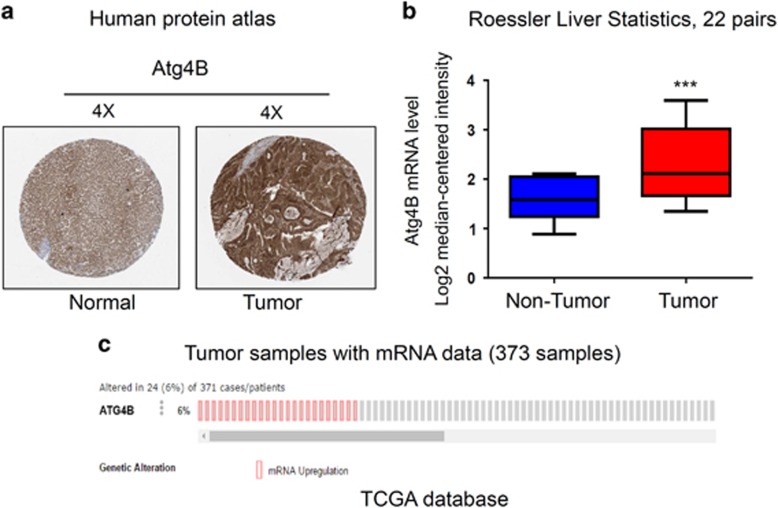
Atg4B is upregulated in human hepatocellular carcinoma specimens. (**a**) Atg4B expression in normal liver tissue and hepatocellular carcinoma specimens. Images were taken from the Human Protein Atlas (http://www.proteinatlas.org) online database. (**b**) Oncomine data showing Atg4B expression in normal *vs* tumor of liver (*n*=22). (**c**) Atg4B mRNA expression in the TCGA liver cancer RNAseq (IlluminaHiSeq; *N*=371) data set.

To determine the clinical relevance of Atg4B expression, we first analyzed the Atg4B protein expression in clinical specimens from the human protein atlas (www.proteinatlas.org). We found that Atg4B had the positive strong expression in HCC, and negative weak expression in normal liver (Figure 6a). Consistently, Atg4B mRNA level was higher in HCC tissues than that in normal liver tissues (0.86±0.1352, vs1.098±0.2247, *P*<0.001, *n*=22) in Roesser liver database (Compendia Biosciences, www.oncomine.org) (Figure 6b) and TCGA liver cancer RNAseq (IlluminaHiSeq; *N*=371) data set (https://genome-cancer.ucsc.edu). (Figure 6c).

## Discussion

Although autophagy has been implicated in resistance to anticancer therapy but its role in this aspect in HCC is not clear, and the underlying mechanism is still not fully understood.^7, 32^ The present demonstrates that Egr-1 has a critical role in modulating IR-induced autophagy and thus radiotherapy resistance in HCC cells. Specifically, suppression of Egr-1 activity abrogates IR-induced autophagy and sensitizes HCC cells to IR treatment. Egr-1 promotes transcription of Atg4B by binding directly to its promoter region at three sites. Importantly, Atg4B is upregulated at both mRNA and protein levels in clinical HCC specimens, suggesting its clinical significance.

Egr-1 is a zinc finger transcription factor that is rapidly induced by a wide range of extracellular stimuli.^33, 34^ Growing evidence indicates that Egr-1 has a key role in controlling many process of diseases, such as hypertension, chronic obstructive pulmonary disease, as well as cancers.^35, 36, 37, 38^ Much like the multifunctional nature of a Swiss army knife, Egr-1 has been demonstrated to play diverse roles in different types of cancers relying on the integrated functions of various genes it regulates.^39, 40, 41^ For instance, Egr-1 promotes the invasion ability of oral squamous cell carcinoma cells by regulating cathepsin D.^42^ Similarly, expression of Egr-1 positively correlated with the progression from non-muscle invasive bladder cancers to muscle invasive bladder cancer.^43^ Paradoxically, Egr-1 function as tumor suppressor in non-small-cell lung carcinoma by regulating KRT18 expression.^40^ In this study, we showed that Egr-1 is rapidly upregulated by IR treatment in HCC cells, leading to the increased cell resistance to IR. Suppression of Egr-1 transcriptional activity by Ad-DN-Egr-1 decreases the cell viability, colony formation and migration ability, as well as sensitizes HCC cells to irradiation therapy (Figures 1 and 2). We further show that autophagy is important to Egr-1-mediated radioresistance. For instance, radiation remarkably induces autophagy of HCC cells. Cell viability assay and western blot results indicate that depletion of autophagy by inhibitor 3-MA or CQ enhances IR-induced apoptotic cell death (Figure 3).

A previous report showed that Egr-1 regulates autophagy in cigarette smoke-induced chronic obstructive pulmonary disease, knockdown of Egr-1 inhibits the expression of Atg4B in Beas-2B cells.^38^ Our recent study also suggests that Egr-1 promotes hypoxia-induced autophagy to enhance chemoresistance.^21^ These studies suggest that Egr-1 may have a critical role in regulation of autophagy, but the molecular mechanism is still not fully understood. To further explore the function of Egr-1 on autophagy caused radioresistance of HCC cells, bioinformatics analysis was performed. Interestingly, the analysis result showed three putative Egr-1-binding motifs present in promoter region of Atg4B, a critical Atg involved in autophagosome formation process (Figure 4a). We further presented evidence that Egr-1 increases Atg4B expression both at mRNA and protein level, suggesting a role of Egr-1 in Atg4B transcription (Figure 4b). Luciferase report assay and ChIP assay performed on SMMC-7721 cells confirmed the binding of Egr-1 with Atg4B promoter *in vitro* and *in vivo* (Figure 4c and d). We also discovered that overexpression of Egr-1 restores the anti-apoptosis and colony-formation ability repressed by 3-MA, CQ or siRNA targeting Atg4B (Figure 5). Specifically, we found the expression of Atg4B is positively strong in HCC specimens compared with normal liver samples (Figure 6).

In summary, our results demonstrated that Egr-1 facilitates HCC cells resistant to radiotherapy partially through modulating of IR-induced autophagy. Our findings strongly suggest that targeting Egr-1 is a therapeutic strategy for HCC.

## Materials and methods

### Cell lines and reagents

The human HCC cell lines HepG2 and SMMC-7721 were obtained from ATCC (USA) and Cancer Cell Repository (Shanghai, China), respectively. Cells were maintained in Dulbecco's Modified Eagle Medium with 10% fetal bovine serum at standard cell culture conditions (37 °C, 5% CO2 in humidified incubator). Antibodies against Egr-1 (#4145), Bcl-2 (#15071), Bax (#5023), cleaved caspase-3 (#9661), Vimentin (#5741), *N*-Cadherin (#13116) and Snail (#3879) were purchased from Cell Signaling Technology (Danvers, MA, USA). The antibody against α-tubulin (A5441) was from Sigma-Aldrich (St Louis, MO, USA). siRNA duplexes specific for Atg4B (siAtg4B, #6336) and a non-targeting siRNA (siCon, #6568) were purchased from Cell Signaling Technology.

### Irradiation

The SMMC-7721 and HepG2 cells were plated in six-well plates and incubated in the culture medium until 70–80% confluence was attained. Cells were irradiated using a 60Co γ-ray facility (Siemens, Oncor Impression at nominal energies of 6 MeV) at room temperature. The doses applied in the experiments varied from 0 to 8 Gy.

### Colony-formation assay

Cells were infected with Ad-GFP, Ad-DN-Egr-1or Ad-Egr-1 and exposed to irradiation at 8 Gy 48 h after infection. After irradiation, cells were trypsinized, resuspended and seeded in six-well plates (1 × 10^3^ cells/well). For autophagy inhibitor treatment, the medium was replaced with fresh medium containing 3-MA or CQ for every 4 days. Surviving colonies were fixed and stained 14 days later.

### Transwell migration assay

Cells were infected with Ad-GFP or Ad-DN-Egr-1 for 48 h and then exposed to irradiation at 8 Gy. After irradiation, cells were trypsinized, resuspended in serum-free Dulbecco's Modified Eagle Medium, and transferred to the upper chamber (5 × 10^3^ cells in 500 μl) (24-well insert, 8-μm pore size, BD Biosciences, San Jose, CA, USA). Dulbecco's Modified Eagle Medium containing 10% fetal bovine serum was added to the lower chamber. After incubation for 16 h, non-invaded cells were removed from the upper wells with cotton swabs, whereas the invaded cells were fixed with 4% paraformaldehyde, stained with 0.1% crystal violet and photographed (× 40) in five independent fields for each well. Each test was repeated in triplicate.

### Promoter reporters and dual luciferase assay

The human Atg4B promoter was amplified by PCR using the following primers: Fwd: 5′- CCGCTCGAGGGCGCCGGCCGGATCGATCG-3′ Rev: 5′- CCCAAGCTTCCATCTTGC- GGTACGGACGT-3′. PCR products were digested and then cloned into the pGL3-Basic vector (Promega, Madison, WI, USA). The Atg4B mutant promoter constructs were generated using the KOD -Plus- Mutagenesis Kit (TOYOBO, Osaka, Japan), all PCR primers for mutation were listed in Supplementary Table S1 and high fidelity enzyme Phusion was used for PCR amplification. All PCR products were verified by DNA sequencing. In the dual luciferase assays, cells were cultured for 36 h after transfection, and cell lysate was used for luciferase assays using the dual luciferase reporter assay system (Promega). Luciferase activities were normalized to the co-transfected pRL-TK plasmid. All experiments were conducted at least twice, in triplicate.

### ChIP assay

ChIP assays were performed using the ChIP assay kit from Cell Signaling Technology according to the manufacturer's protocol. The resulting precipitated DNA samples were analyzed by PCR using primers (Supplementary Table S1). For instance, primers Atg4B-ChIP-5.1 and Atg4B-ChIP-3.1 would amplify a 180 bp region (EBS1) of human Atg4B promoter, and Atg4B-ChIP-5.2A and Atg4B-ChIP-3.2A amplify a 190 bp region (EBS2), and Atg4B-ChIP-5.3 and Atg4B-ChIP-3.3 amplify a 300 bp region (EBS3). The PCR products were resolved electrophoretically on a 2% agarose gel and visualized by ethidium bromide staining.

### siRNA transfection

Double-stranded siRNA were used to knockdown Atg4B from cells at a final concentration of 100 nm. SMMC-7721 and HepG2 cells were transiently transfected with siCon or siAtg4B using Lipofectamine 2000 (Invitrogen, Carlsbad, CA, USA) according to the vendor's instruction.

### Real-time PCR

Total RNA was extracted from cultured cells using the Trizol reagent (Invitrogen). Real-time RT-PCR assays were performed as described previously.^44^ GAPDH was used as an internal control. All experiments were performed in triplicate.

### Western blotting

Cells were harvested in sample buffer (62.5 mmol/l Tris-HCl (pH 6.8), 10% glycerol and 2% sodium dodecyl sulfate) and boiled for 5 min. Standard western blot analysis of cell lysates was carried out using the antibodies described earlier.

### Analysis of Atg4B mRNA and protein expression in human HCC

Atg4B protein expression in HCC tissues and normal tissues was determined from the human protein atlas (www.proteinatlas.org). HCC Atg4B gene expression was determined through analysis of Roessler and TCGA databases, which are available through Oncomine (Compendia Biosciences, www.oncomine.org) and UCSC (https://genome-cancer.ucsc.edu). High and low groups were defined as above and below the mean respectively.

### Statistical analysis

Data are presented as mean±s.e.; the Student's *t*-test was used for assessing the difference between individual groups and *P*⩽0.05 was considered statistically significant.
