# Supplementary material for: Egr-1 regulates irradiation-induced autophagy through Atg4B to promote radioresistance in hepatocellular carcinoma cells
Source: Oncogenesis. 2017 Jan 30;6(1):e292–. doi: 10.1038/oncsis.2016.91 (PMC5294254; doi:10.1038/oncsis.2016.91)
Supplement: Supplementary Information [file oncsis201691x1.docx]

**Supplementary information, Table S1** **Primers used in this study**

**Atg4B promoter cloning primers**

Atg4B–Luc-5.2 CCGCTCGAGGGCGCCGGCCGGATCGATCG

Atg4B–Luc-3.2 CCCAAGCTTCCATCTTGCGGTACGGACGT

**Mutation primers**

Mut-1-5.1 GCCCGAAAACGCCCCAGCCCCCGCC

Mut-1-3.1 CCGCTGCGGGCGCCTGCGCCTCCAT

Mut-2-5.1 CGGCGAAAACGCCCCCCGCGTTCGG

Mut-2-3.1 GGCCTCGGCGGCCGGGGCGCGGGGG

Mut-3-5.1 AGTCGAAAACGGCGGGGCAAGTCCG

Mut-3-3.1 ACTCCCCCGCCCGTCTGCCCGCCTG

**ChIP primers**

Atg4B-ChIP-5.1 AACTGCTCCTGGGTCCTCTA

Atg4B-ChIP-3.1 AGGCGGCGAAGACGATACGG

Atg4B-ChIP-5.2A TACGGCAAGATGGAGGCGCAGGC

Atg4B-ChIP-3.2A ATGTCGCGGTACGGACTTGCCCC

Atg4B-ChIP-5.3 [G](javascript:__.opf('f1081'))GCGCCGGCCGGATCGATCG

Atg4B-ChIP-3.3 ATCTGCTGTGCGGCCGTGAA
